# Severe Traumatic Brain Injury and Ventriculoperitoneal Shunt Challenges: A Case Report

**DOI:** 10.7759/cureus.35828

**Published:** 2023-03-06

**Authors:** Lucie Cunha, Eduarda Pereira

**Affiliations:** 1 Intensive Care Unit, Centro Hospitalar Universitário do Algarve - Portimão, Portimão, PRT; 2 Neurocritical Care Unit, Intensive Care Department, Hospital São João, Porto, PRT

**Keywords:** cranioplasty, brain herniation, hydrocephalus, ventriculoperitoneal shunt, traumatic brain injury

## Abstract

Traumatic brain injury (TBI) is an acquired damage to the brain caused by an external mechanical force and may result in temporary or long-term disability. It is a prevalent condition that highlights the need for increased awareness. Despite advances in the technology used to monitor and guide therapy, it is a difficult and complex disease process to manage. Elevated intracranial pressure (ICP), a complication of neurologic injury, is associated with increased mortality and worse outcomes. Decompressive craniectomy is effective in controlling ICP and is potentially lifesaving in patients who have failed medical therapy. As a long-term treatment, implanting a ventricular-peritoneal (VP) shunt is the typical neurosurgery method for treating hydrocephalus following TBI. Nevertheless, under certain circumstances, VP shunts fail and an interdisciplinary approach is essential to improve patients’ outcomes.

Herein, we present the case of a previously healthy young male patient who successfully underwent a surgical treatment following TBI. The aim of this case report is to share the knowledge gained at Centro Hospitalar Universitário do São João, in Portugal, regarding complications of VP shunts and how cranioplasty aided in the resolution of the problem.

## Introduction

Traumatic brain injury (TBI) is described as a change in brain function, or other signs of brain disease, induced by an external force [1]. It is characterized as any period of a diminished level of awareness or alteration in mental state, any loss of recent memory, or neurologic deficits. Although clinical judgment is applied to determine if the patient's symptoms are a result of the TBI, these do not rule out the diagnosis of TBI in the face of complicating circumstances like alcohol intoxication, recreational drug use, or medical disease (pain, post-traumatic shock, or medication). Furthermore, this definition can incorporate imaging or laboratory investigations [2].

Despite being a silent epidemic, it is a serious public health and socio-economic concern around the world, causing more death and disability than any other traumatic injury. Worldwide, there is a prevalence of 759 per 100,000 people and an annual incidence between 27 and 69 million [3-5].

The degree of brain injury can be classified as mild, moderate, or severe, depending on the resulting symptoms of the TBI. To quantify the level of consciousness after TBI, it is common to use physical exam findings and grades according to the Glasgow Coma Scale (GCS). A person's GCS score can range from 3 (completely unresponsive) to 15 (fully awake) [6].

The greatest impact on morbidity and mortality of surviving TBI patients depends on the early recognition of the disease since initial management is the most critical phase. Once the trauma has occurred, there is nothing that can be done to reverse the initial brain harm. As so, the mainstay of action is aimed at stabilizing an individual with TBI and limiting secondary brain injury. Nearly half of the patients with severe head injuries will require surgery [1].

## Case presentation

We present the case of a 38-year-old male who was previously healthy but was involved in a road accident. The victim was without a helmet at the time of the injury and was found with an initial GCS of 14 (eyes: 4, verbal: 4, motor: 6) associated with agitation and headache. The pupil size and light reflex were intact and the neurologic examination demonstrated no focal neurologic deficit. He had right otorrhagia, a left occipital blunt wound, and abrasive lesions in the left nasal and zygomatic region. The patient was hemodynamically stable on admission, with a blood pressure of 162/88 mmHg, a heart rate of 68 beats per minute, and 99% oxygen saturation. A head computerized tomography (CT) scan revealed sulcal subarachnoid hemorrhage and multiple cerebral contusions. Due to evidence of elevated intracranial pressure (ICP) at 26 mmHg, the patient underwent a decompressive craniectomy. Despite medical measures (osmotic therapy and diuresis with hypertonic saline bolus and mannitol), a persistent increased ICP motivated a second surgery 48 hours later to enlarge the borders of the previous craniectomy, and an external ventricular drain was placed. Then, the patient developed a cerebrospinal fluid leak through the surgical wound, which led to a subdural hygroma complicated by *Staphylococcus epidermidis* and *Enterobacter aerogenes* meningitis. Surgical debridement, subdural drain, and direct pressure were done, associated with a 21-day course of antibiotic therapy with meropenem and vancomycin with a favorable evolution.

**Figure 1:**
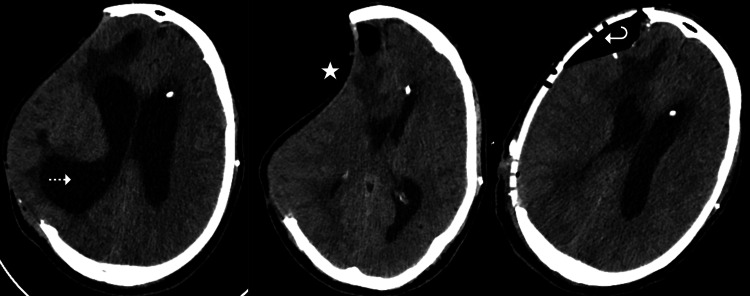
Axial view brain CT scan Dashed arrow shows hydrocephalus; star shows paradoxical brain herniation; curved arrow shows cranioplasty with no complications associated

Fifty-five days after the accident, a ventriculoperitoneal (VP) Polaris® adjustable valve (Sophysa, Orsay, France) was inserted. The VP shunt placement surgery was uneventful, however, the valve position and, therefore, pressure (confirmed by radiological images) was a challenge. The programmable valve allows pressure adjustments without reoperation and, initially, the valve was set at position 3 (100 mmH_2_O median pressure). Twenty-four hours after the surgery, the patient developed a sinking flap skin, vomiting, and progressive unconsciousness with a Glasgow Coma Scale (GCS) of 12 (eyes: 3, verbal: 3, motor: 6). Head CTscan showed paradoxical brain herniation and midline shift to the left by 13.5 mm. Conservative management, including preservation of supine bed rest and fluid therapy, was initiated. The valve position was also augmented to 4 (150 mmH_2_O median pressure). However, these measures were insufficient, since the patient continued to deteriorate clinically. To counterbalance this, the valve was adjusted to position 5 (200 mmH_2_O median pressure) and 24hours hours later, the patient complained of headache and extremity weakness, and the head CT scan showed hydrocephalus. In the next five days, the valve position was adjusted between positions 4 and 5 multiple times. It was only normalized when a cranioplasty with a customized polyether ether ketone implant was placed and the valve pressure was set at position 4 (150 mmH_2_O median pressure) and tolerated (Figure 1). This happened 67 days after the accident (12 days after the insertion of the VP shunt).

The patient survived this prolonged hospital stay (77 days of hospitalization) and was discharged to a rehabilitation unit. The first follow-up examination was scheduled three months after hospital discharge. At this point, the patient had his VP shunt at position 4 (150 mmH2O median pressure) and good clinical and imaging evolution. Six months after the accident, he had a GCS of 14 (eyes: 4, verbal: 4, motor: 6) because of the aphasia and some motor impairment with diminished muscle strength in the left upper and lower limbs (grade 4 in 5 according to the Medical Research Council scale). He walked with the support of a Canadian crutch but was independent in activities of daily living.

## Discussion

A young male who was involved in a car accident was admitted to the hospital and TBI was diagnosed. An extensive workup was performed to control elevated intracranial pressure (ICP) and several complications were solved during his hospitalization. 

The severity of TBI can be classified into mild, moderate, and severe. In patients with severe TBI, cerebral autoregulation can be disrupted. Elevated ICP is a medical emergency and treatment should be undertaken as expeditiously as possible. At first, a medical and pharmacological approach (head elevation, hyperventilation, and osmotic therapy) should be attempted. When previous attitudes proved to be futile, more aggressive ones are needed such as decompressive craniectomy. This surgery is effective and is potentially lifesaving in patients who fail more conservative management. Still, the prognosis of surgical interventions differs from the situation prior to hospitalization. The individual’s age and health in association with the location of the wound will outline the disabilities resulting from the TBI. Additionally, this patient developed meningitis, and antibiotic therapy with surgical debridement was done, extending his hospitalization.

An obstruction of the cerebrospinal fluid flow can cause Hydrocephalus and the placement of a shunt (VP, ventriculoatrial, or lumboperitoneal) is often necessary for treatment. The use of programmable shunt devices has many advantages due to their adjustable-pressure valve system and the capacity to percutaneously adjust cerebrospinal fluid pressure-related flow depending on the patient’s clinical response or development of subdural effusions [4,7,8]. However, the pressure control device used in this case had only five ranges available (from position 1 to position 5). Since he developed complications in various settings, it was particularly difficult to manage this patient. The pressure and fluid dynamics caused by the cranioplasty were advantageous and made it possible to transfer the patient from a neurointensive care unit to a neurosurgery ward.

This article aims to raise awareness about the detection and management of the possible complications of VP valves.

## Conclusions

As a major health problem, TBI is a critical priority. There are many clinical cases described in the literature of complications associated with the placement of a VP shunt. This case shows a patient who had a VP shunt placed and how, in cooperation with neurosurgery, it was a challenge to establish the valve pressure with which the patient was comfortable. Cranioplasty was essential for the case to be successful. The management of TBI is challenging, however, prognosis and recovery can be more difficult than the treatment itself. Therefore, a personalized and multidisciplinary approach should be taken.
